# Supplementary material for: Picasso-server: a community-based, open-source processing framework for super-resolution data
Source: Commun Biol. 2022 Sep 8;5:930. doi: 10.1038/s42003-022-03909-5 (PMC9458736; doi:10.1038/s42003-022-03909-5)
Supplement: Supplementary file 2 — Supplementary Information [file 42003_2022_3909_MOESM2_ESM.pdf]

Supplementary Note 1

Picasso-Server: Code Availability & Installation

Installation Instructions

- The latest release can be found here: https://github.com/jungmannlab/picasso/releases.
  This contains a build for a one-click installer for Windows
- The release no for the manuscript is 0.4.11 and can be found here: https://github.com/jungmannlab/picasso/releases/tag/v.0.4.11
- Picasso is on the Python Package Index and can be installed via pip: “pip install picassosr”.
  - Note that for mac systems (e.g. the M1 series), you might need to install PyQt separately; see the instructions on the link below
  - Launching Picasso for the first time might take much longer (> 10s) than when launching it again
- For additional and more extensive Installation instructions, see the readme: https://github.com/jungmannlab/picasso/blob/master/readme.rst

Tutorial Video

- A 9:56 minute demonstration video can be found here:
  - https://vimeo.com/737363377
  - Note the video shows several version numbers but is valid up to version 0.4.11
- It covers the following aspects:
  - Download & Installation
  - Localization
  - Render
  - Database
  - Watcher
- The following raw files were used in the demonstration video (Tubular structure 2D):
  - https://srm.epfl.ch/Challenge/ChallengeSimulatedData

Documentation

- Additional documentation on usage of the server module can be found here: https://picassosr.readthedocs.io/en/latest/server.html
- More documentation on the Watcher:
  - https://picassosr.readthedocs.io/en/latest/server.html#watcher
- Cross-Platform-Support to run Picasso Server with Docker:
  - https://picassosr.readthedocs.io/en/latest/server.html#docker

Code

- Picasso-Server is integrated into the main Picasso codebase at https://github.com/jungmannlab/picasso.
- The server-specific codebase can be found here: https://github.com/jungmannlab/picasso/tree/master/picasso/server.

Supplementary Note 2

Picasso Documentation

Release 0.4.11

Maximilian Thomas Strauss

Aug 09, 2022

Contents:

1 design **3**
1.1 Design rectangular DNA origami . . . 3

2 simulate **5**
2.1 Simulate DNA-PAINT image acquistions . . . 6

3 server **9**
3.1 Functionality . . . 9
3.2 Database . . . 9
3.3 Localize . . . 9
3.4 Server . . . 10

4 localize **15**
4.1 Identification and fitting of single-molecule spots . . . 16
4.2 Camera Config . . . 16
4.3 3D-Calibration . . . 18

5 filter **21**
5.1 Filtering of localizations . . . 21

6 render **23**
6.1 Opening Files . . . 23
6.2 Drift Correction . . . 24
6.3 Picking of regions of interest . . . 25
6.4 Dialogs . . . 25
6.5 Menu items . . . 27

7 average **35**
7.1 Particle Averaging . . . 35

8 filetypes **37**
8.1 Movie Files . . . 37
8.2 HDF5 Files . . . 37
8.3 Importing HDF5 files in Pandas, MATLAB and Origin . . . 37
8.4 Localization HDF5 Files . . . 38
8.5 HDF5 Pick Property Files . . . 40
8.6 YAML Metadata Files . . . 40

9 CMD **41**
9.1 localize . . . 41
9.2 csv2hdf . . . 42
9.3 join . . . 42
9.4 link . . . 43
9.5 cluster_combine . . . 43
9.6 cluster_combine_dist . . . 43
9.7 clusterfilter . . . 43
9.8 undrift . . . 43
9.9 density . . . 43
9.10 dbscan . . . 43
9.11 hdbscan . . . 43
9.12 dark . . . 43
9.13 align . . . 43
9.14 groupprops . . . 44
9.15 pc . . . 44
9.16 nneighbor . . . 44
9.17 render . . . 44
9.18 design . . . 44
9.19 simulate . . . 44
9.20 average . . . 44
9.21 average3 . . . 44

10 Postprocessing **45**
10.1 Jupyter Notebooks . . . 45

11 nanoTRON **47**
11.1 nanoTRON Train . . . 47
11.2 nanoTRON Predict . . . 48

12 FAQ **49**
12.1 Localize . . . 49

13 Indices and tables **51**

This documentation is based on the Nature Protocols publication. Its aim is to cover all the recent updates.

CHAPTER 1

design

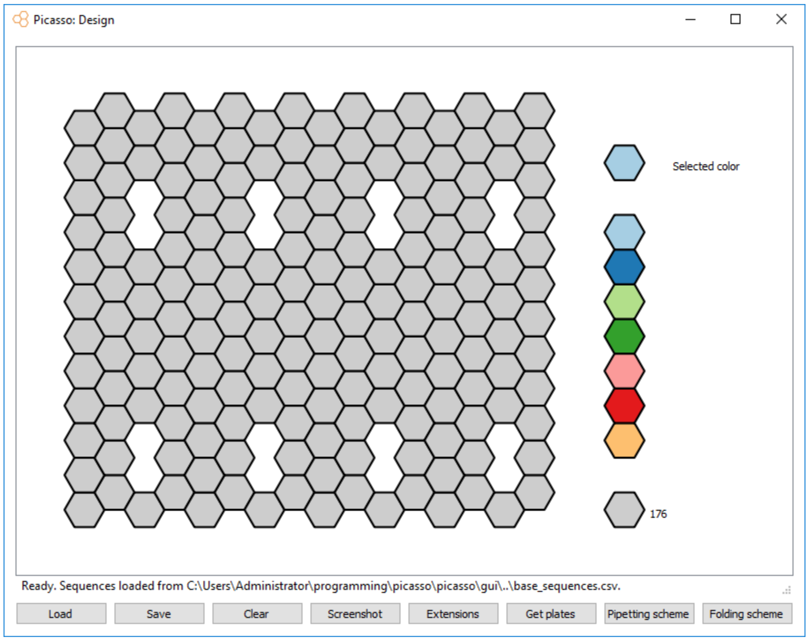

1.1 Design rectangular DNA origami

- Start the design module. A canvas with hexagons, corresponding to the flat origami sheet will appear.
- Design a pattern of DNA-PAINT binding sites by clicking on the hexagons.
- Select a color by clicking on the color palette on the right side.
- Each color corresponds to a different extension that can be defined later.
- The default state of a hexagon (gray color) indicates that this staple will not be extended. Click on the `Clear`-Button to reset all hexagons.

- Some hexagons are marked with a `V` after coloring. This indicates that this staple, in contrast to all the others is pointing downwards at its 3'-end
- Click on "Extensions" to define the sequence for each extension.
- Use `Get plates` to get an excel sheet with all needed sequences in a plate format.
- Use `Pipetting scheme` and select the folder with your plate list to generate a pipetting scheme.
- Use `Folding scheme` to get a table for pipetting folding mixes
- A design can be saved and loaded using the `Save` and `Load`-Buttons.
- A saved design can also be loaded into the `Picasso: Simulate` module.

CHAPTER 2

simulate

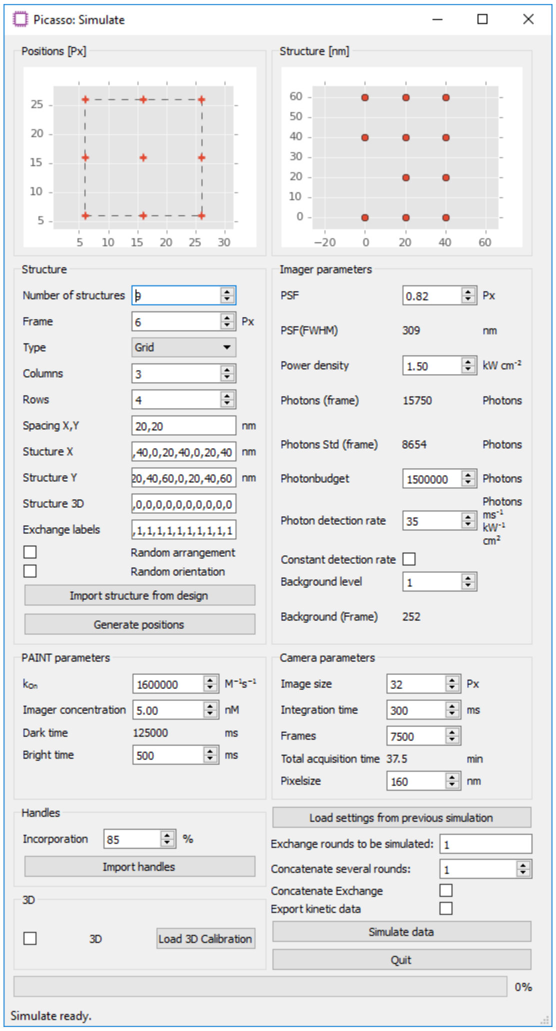

Picasso's simulation module (`Picasso: Simulate`) is a tool for evaluating experimental conditions for DNA-PAINT and generating ground-truth data for test purposes. This allows systematic analysis of how different experimental parameters such as imager concentration, target density or integration time influence the imaging quality and whether the target structure can be resolved with DNA-PAINT. By default, `Picasso: Simulate` starts with pre-set parameters that are typical for a DNA-PAINT experiment. Thus, meaningful raw DNA-PAINT data can be readily simulated for a given input structure without the need of a super-resolution microscope. The simulation output is a movie file in .raw format, as it would be generated during an in vitro DNA-PAINT experiment on a microscope.

2.1 Simulate DNA-PAINT image acquistions

1. Start `Picasso: Simulate`.
2. Define the number and type of structures that should be simulated in the group `Structure`. Predefined grid- and circle-like structures can be readily defined by their number of columns and rows, or their diameter and the number of handles, respectively. Alternatively, a custom structure can be defined in an arbitrary coordinate system. To do so, enter comma-separated coordinates into `Structure X` and `Structure Y`. The unit of length of the respective axes can be changed by setting the spacing in `Spacing X, Y`. For each coordinate point, an identifier for the docking site sequence needs to be set in `Exchange labels` as a comma-separated list. Correctly defined points will be updated live in the `Structure [nm]` window. Note that entries with missing x coordinate, y coordinate or exchange label will be disregarded. When a structure has been previously designed with `Picasso: Design`, it can be imported with `Import structure from design`. A probability for the presence of a handle can be set with `Incorporation`. By default, all structures are arranged on a grid with boundaries defined by `Image size` in `Camera parameters` and the `Frame` parameter in the `Structure` group. `Random arrangement` distributes the structures randomly within that area, whereas `Random orientation` rotates the structures randomly. Selecting the button `Generate positions` will generate a list of positions with the current settings and update the preview panels. A preview of the arrangement of all structures is shown in `Positions [Px]`, whereas an individual structure is shown in `Structure [nm]`.
3. The group `PAINT Parameters` allows adjustment of the duty cycle of the DNA-PAINT imaging system. The mean dark time is calculated by τd = 1/(kon·c). The mean ON time in a DNA-PAINT system is dependent on the DNA duplex properties. For typical 9-bp imager/docking interactions, the ON time is ~500 ms.
4. In `Imager Parameters`, fluorophore characteristics such as PSF width and photon budget can be set. Adjusting the `Power density` field affects the simulation analogously to changing the laser power in an experiment.
5. The `Camera parameters` group allows the user to set the number of acquisition frames and integration time. The default image size is set to 32 pixels. As the computation time increases considerably with image size, it is recommended to simulate only a subset of the actual camera field of view.
6. Select `Simulate data` to start the simulation. The simulation will begin by calculating the photons for each handle site of every structure and then converting it to a movie that will be saved as a .raw file, ready for subsequent localization. All simulation settings are saved and can be loaded at a later time with `Load from previous simulation`.
7. (Optional step for multiplexing) Multiplexed Exchange-PAINT data can be simulated by adjusting the `Exchange Labels` setting. For each handle in the custom coordinate system (`Structure X`, `Structure Y`), an Exchange round can be specified. The different imaging rounds can be visually identified by color in the `Structure [nm]` figure. For each round, a new movie file will be generated. By default, the simulation software detects the number of exchange rounds based on the structure definition and will simulate all multiplexing rounds with the same imaging parameters. It is possible to have different imaging parameters for each round, e.g. when using image s with different ON-times. To do so, one can simulate multiplexing rounds individually. In the `Exchange rounds to be simulated` field, enter only the rounds that should be simulated with

the current set of parameters. Change the set parameters and the multiplexing round and simulate the next data sets. Repeat until all multiplexing rounds are simulated.

CHAPTER 3

server

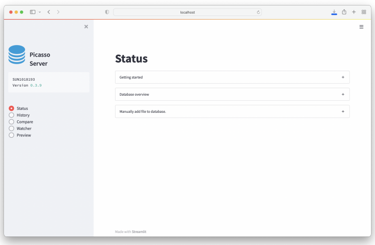

3.1 Functionality

Picasso Server allows to continuously track performance metrics of your super-resolution experiments. It does so by recording the metadata, derived summary statistics, and results of post-processing algorithms (such as the NeNA value) to a local SQL database. The database can be interactively explored via a browser application.

You can also set up a `Watcher` that allows to continuously process new files in a folder.

3.2 Database

The local SQL database will be stored in the `.picasso` folder in your home directory. The path is also displayed in the *Getting started*-tab in the *Status* page. To directly access the database the tool DB Browser for SQLite is recommended.

3.3 Localize

The integration within `Localize` is by pressing the `Estimate-` button in the `Sample Quality` field in `Parameters`. The button can be pressed once the image stack has been localized. It calculates Localizations per

Frame, NeNA, Drift and bright time based on a subset of the data (i.e. max. 1 Mio localizations). The estimate and additional summary statistics will then be stored in the local database.

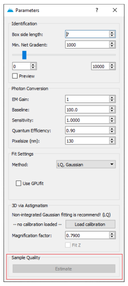

3.4 Server

Server is a tool to interactively explore the database and to enable continuous processing workflows. When launching server, i.e. clicking on the shortcut or executing the Python command `picasso server`, the command line will start and show you the local IP address on where the server is running. When launching picasso server, it should open your default browser automatically and redirect to the Picasso Server page. You can also use this IP to e.g., connect to `Picasso Server` from within a network. For this, take the `Network URL` that is displayed in the command line and access it via browser.

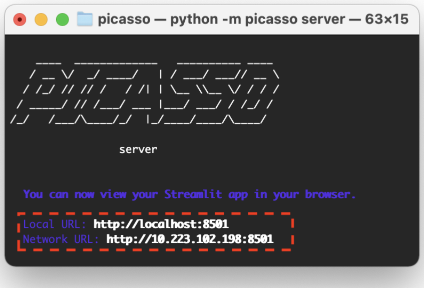

In case you close the website tab or the browser, `Picasso Server` will run in the background until the command line window is closed. You can go back to the website by re-entering the URL.

3.4.1 Status

Displays the current database status and documentation. The page also has a menu called `Manually add file to database.`. Here, you can add folders with already processed files to the database.

3.4.2 History

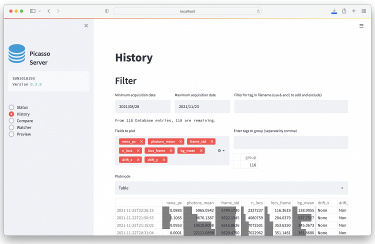

Explore summary statistics of processed files. It is possible to filter by filename and group. The following modes of display exist:

- Table: A table of the results. Each field contains a barplot showing the value relative to the column's maximum.
- Scatter: Scatterplot of results. This also allows to draw trendlines
- Boxplot: Daily Boxplot

3.4.3 Compare

Compare two files against each other.

The database will store the file path when it is localized. If the file is moved, it will not be selectable.

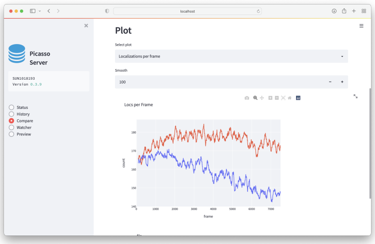

To compare experiments, select one or multiple experiments from the dropdown menu. If multiple hdf files are present, you can select hdf files that belong to the same file family.

Comparing files will load the entire hdf file and could mean that one is comparing millions of localizations. Creating the plots might, therefore, not be instantaneous.

- Localizations per frame: this will plot the localization per frame. This is useful to inspect the performance of an experiment over time.
- Histogram: creates a histogram for the population. This is useful e.g., for comparing background signals.

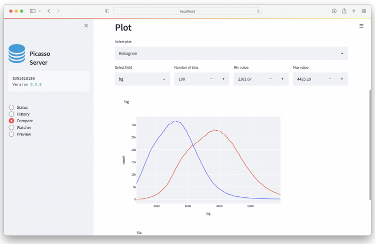

3.4.4 Watcher

- Set up a file watcher to process files in a folder with pre-defined settings automatically.
- All new files and raw files that aren't yet in the database will be processed.
- You can define different parameter groups so that a file will be processed with different settings.
- You can also chain custom commands to the watcher.
- The watcher will check for the following filetypes: ('.raw', '.ome.tif', '.ims')
- The watcher will be able to process consecutive files created with MicroManager (e.g. *Pos0*, *Pos0_1*, *Pos0_2*) if they contain *MMStack_Pos0* in the filename

Logging

Each watcher will write their statust to a logfile. The path is visible when setting up the logfile and when checking the currently running watchers. To view the log, select the logfile and expand the *Log*-field.

Multiple Parameter Groups

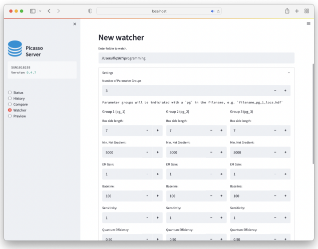

With multiple parameter groups a file will be processed with different settings. To make multiple parameter groups available, set the *Number of Parameter Groups* to a value larger than 1. The file ending will be different according to the parameter group used. E.g. if you have two parameter groups, file *testfile.raw* will be processed as *testfile_pg_1_locs.hdf5* and *testfile_pg_2_locs.hdf5*.

Custom commands

You can use the "Custom command" to execute a custom script after a file was processed. Consider the following example for a script that you want to execute named test.py::

```
import sys
from slack_sdk.webhook import WebhookClient
url = "REPLACE_WITH_SLACKHOOK"
webhook = WebhookClient(url)

_, filename = sys.argv[0], sys.argv[1]

response = webhook.send(text=f"Processed file {filename}!")
```

This script would send a message to a slack webhook with the first argument as filename. To call this from the watcher, we need to point to a python environment. E.g. for a conda installation at `C:\ProgramData\Miniconda3\python.exe` and the script being located at `C:\Users\Maximilian\Desktop\test.py` the complete command to enter in Picasso server would

be: `C:\ProgramData\Miniconda3\python.exe C:\Users\Maximilian\Desktop\test.py $FILENAME`.

When having an existing Picasso one-click installation, Picasso can directly be called via the command-line. Some example commands would then be:

- `picasso undrift $FILENAME` for drift correction
- `picasso link $FILENAME` for linking localizations
- `picasso dbscan $FILENAME 0.1 2` for performing DBSCAN cluster analysis with 0.1 / 2

3.4.5 Preview

Preview will render the super-resolution data in the browser.

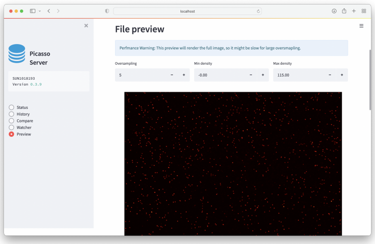

The database will store the file path when it is localized. If the file is moved, it will not be selectable.

3.4.6 Docker

If you want to install picasso server in a headless linux or mac system, the provided dockerfile might be useful for installation. * Build the docker image from the dockerfile (clone the github repository): `docker build -t picasso .` * Run the docker image (interactive mode, port forwarding and with a mounted drive): `docker run -it -p 8501:8501 --volume "C:/Users/Maximilian/Desktop/data:/home/picasso/data" picasso` Note that you need to replace the respective paths. * Launch picasso server in the docker image: `python3 -m picasso server`

CHAPTER 4

localize

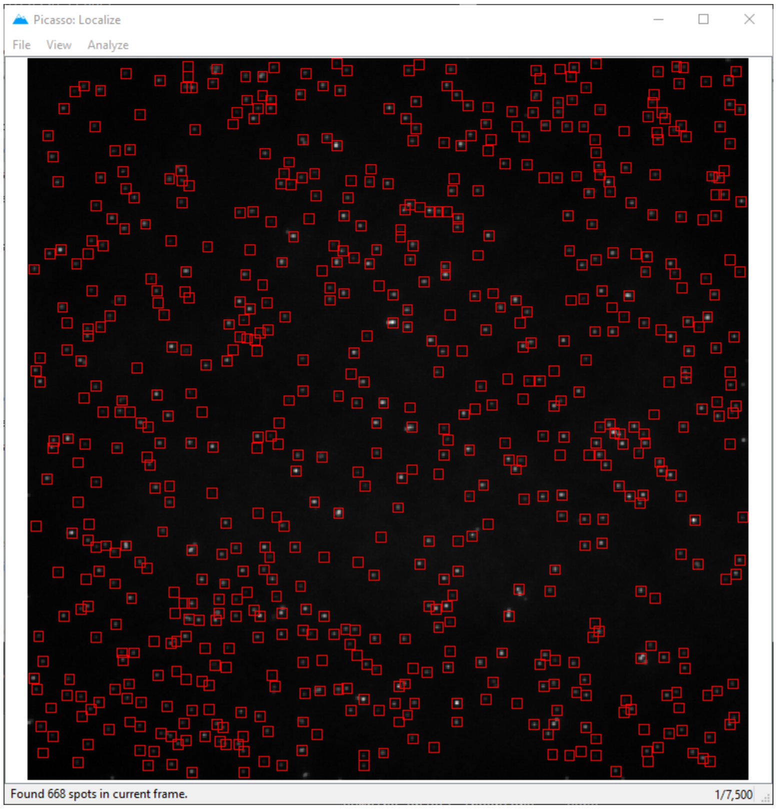

Localize allows performing super-resolution reconstruction of image stacks. For spot detection, a gradient-based approach is used. For Fitting, the following algorithms are implemented:

- MLE, integrated Gaussian (based on Smith et al., 2014.)
- LQ, Gaussian (least squares)
- Average of ROI

4.1 Identification and fitting of single-molecule spots

1. In `Picasso: Localize`, open a movie file by dragging the file into the window or by selecting `File` > `Open movie`. If the movie is split into multiple μManager .tif files, open only the first file. Picasso will automatically detect the remaining files according to their file names. When opening a .raw file, a dialog will appear for file specifications. When opening an IMS file it should be displayed immediately in the localize window. When opening an IMS file with multiple channels, a dialog window will appear allowing you to select the channel that should be loaded. You can navigate through the file using the arrow keys on your keyboard. The current frame is displayed in the lower right corner.
2. Adjust the image contrast (select `View` > `Contrast`) so that the single-molecule spots are clearly visible.
3. To adjust spot identification and fit parameters, open the `Parameters` dialog (select `Analyze` > `Parameters`).
4. In the `Identification` group, set the `Box side length` to the rounded integer value of $6 \times \sigma + 1$, where σ is the standard deviation of the PSF. In an optimized microscope setup, σ is one pixel, and the respective `Box side length` should be set to 7. The value of `Min. net gradient` specifies a minimum threshold above which spots should be considered for fitting. The net gradient value of a spot is roughly proportional to its intensity, independent of its local background. By checking `Preview`, the spots identified with the current settings will be marked in the displayed frame. Adjust `Min. net gradient` to a value at which only spots are detected (no background).
5. In the `Photon conversion` group, adjust `EM Gain`, `Baseline`, `Sensitivity` and `Quantum Efficiency` according to your camera specifications and the experimental conditions. Set `EM Gain` to 1 for conventional output amplification. `Baseline` is the average dark camera count. `Sensitivity` is the conversion factor (electrons per analog-to-digital (A/D) count) and `Quantum Efficiency` should be set according to the average emission wavelength. These parameters are critical to converting camera counts to photons correctly. The quality of the upcoming maximum likelihood fit strongly depends on a Poisson photon noise model, and thus on the absolute photon count. For simulated data, generated with `Picasso: Simulate`, set the parameters as follows: `EM Gain` = 1, `Baseline` = 0, `Sensitivity` = 1, `Quantum Efficiency` = 1.
6. From the menu bar, select `Analyze` > `Localize (Identify & Fit)` to start spot identification and fitting in all movie frames. The status of this computation is displayed in the window's status bar. After completion, the fit results will be saved in a new file in the same folder as the movie, in which the filename is the base name of the movie file with the extension `_locs.hdf5`. Furthermore, information about the movie and analysis procedure will be saved in an accompanying file with the extension `_locs.yaml`; this file can be inspected using a text editor.

4.2 Camera Config

Picasso can remember default cameras and will use saved camera parameters. In order to use camera configs, create a file named `config.yaml` in the picasso folder. To start with a template, modify `config_template.yaml` that can be found in the folder per default. Picasso will compare the entries with Micro-Manager-Metadata and match the sensitivity values. If no matching entries can be found (e.g., if the file was not created with Micro-Manager) the config file will still be used to create a dropdown menu to select the different categories. The camera config can also be used to define a default camera that will always be used. Indentions are used for definitions.

4.2.1 Example 1: Default Camera

```
Cameras:
  Camera1:
```

(continues on next page)

(continued from previous page)

```
    Baseline: 100
    Sensitivity: 0.5
    Quantum Efficiency: 1.0
```

If there is only one camera entry, picasso will create a dropdown menu that has always selected this camera. ### Example 2: Default Camera with different settings Consider a camera that is used for different wavelengths and has different quantum efficiencies:

```
Cameras:
  Camera1:
    Baseline: 100
    Sensitivity: 1
    Quantum Efficiency:
      525: 0.5
      595: 0.6
      700: 0.7
```

This will create a dropdown menu labeled "Emission Wavelength" were the user can select the corresponding entry that will change the sensitivity parameter.

Picasso will search for the following entries in the config.yaml:

Gain

If the string `Gain Property Name` can be found in the config, picasso will search for a value for this key in the Micro-Manager metadata and match if found.

Sensitivity

If the string `Sensitivity Categories` can be found in the config, picasso will create a dropdown menu for each entry, and if the property can be located in the Micro-Manager Metadata, it will be automatically set.

```
Cameras:
  Camera1:
    Baseline: 100
    Quantum Efficiency:
      525: 0.5
    Sensitivity Categories:
      - PixelReadoutRate
      - Sensitivity/DynamicRange
    Sensitivity:
      540 MHz - fastest readout:
        12-bit (high well capacity): 7.18
        12-bit (low noise): 0.29
        16-bit (low noise & high well capacity): 0.46
      200 MHz - lowest noise:
        12-bit (high well capacity): 7.0
        12-bit (low noise): 0.26
        16-bit (low noise & high well capacity): 0.45
```

Here, two Sensitivity Categories are given `PixelReadoutRate` and `Sensitivity/DynamicRange`. In the upper dropdown menu, one now will be able to choose from `540 MHz - fastest readout` and `200 MHz - lowest noise`. Within 540 MHz it will be `12-bit (high well capacity): 7.18`, `12-bit (low noise): 0.29` and `16-bit (low noise & high well capacity): 0.46`. Accordingly for the 200 MHz entry. The dropdown menus can be further nested, e.g., when considering Gain modes:

```
Sensitivity:
  Electron Multiplying:
    17.000 MHz:
      Gain 1: 15.9
      Gain 2: 9.34
      Gain 3: 5.32
```

Quantum Efficiency

If the string `Quantum Efficiency` can be found in the config, picasso will search for a value for the key named `Channel Device` in the Micro-Manager metadata and match if found.

```
Cameras:
  Camera_1:
    Baseline: 100
    Quantum Efficiency:
      525: 0.5
      595: 0.6
      700: 0.7
    Channel Device:
      Name: TIFilterBlock1-Label
      Emission Wavelengths:
        1-R640: 700
        2-G561: 595
        3-B489: 525
    Sensitivity: 0.47
```

Picasso will search for the entry `TIFilterBlock1-Label` in the Micro-Manager Metadata. If this would be `1-G561`, the Emission-Wavelength of `595` will be used to determine the Quantum Efficiency (here 0.6).

Several Cameras

```
Cameras:
  Camera1:
  Camera2:
  Camera3:
```

Once there are several cameras present, Picasso will select the camera who's name matches the Micro-Manager Metadata. If no camera is found, the first one is automatically selected.

4.3 3D-Calibration

4.3.1 Theory

3D Calibration is performed by an adapted version of Huang et al., 2008.

4.3.2 Calibrating z

After entering the step size, picasso will calculate the mean and the variance for sigma_x and sigma_y for each z position. Localizations that are not within one standard deviation are discarded. A six-degree polynomial is fitted to the mean values of x and y.

- mean_sx = cx[6]z0 + cx[5]z1 .. + cx[0]z6
- mean_sy = cy[6]z0 + cy[5]z1 .. + cy[0]z6

The calibration coefficients are stored in the YAML file and contain the parameters of cx and cy. The first entry being c[0], the last being c[6].

4.3.3 Fitting z

For each localization, sigma_x and sigma_y is determined. Similar to the Science paper, the following equation is used to minimize the Distance D: `D = (sx0.5 - wx0.5)^2 + (sy0.5 - wy0.5)^2` with w being `c[6]z0 + c[5]z1 .. + c[0]z6`.

CHAPTER 5

filter

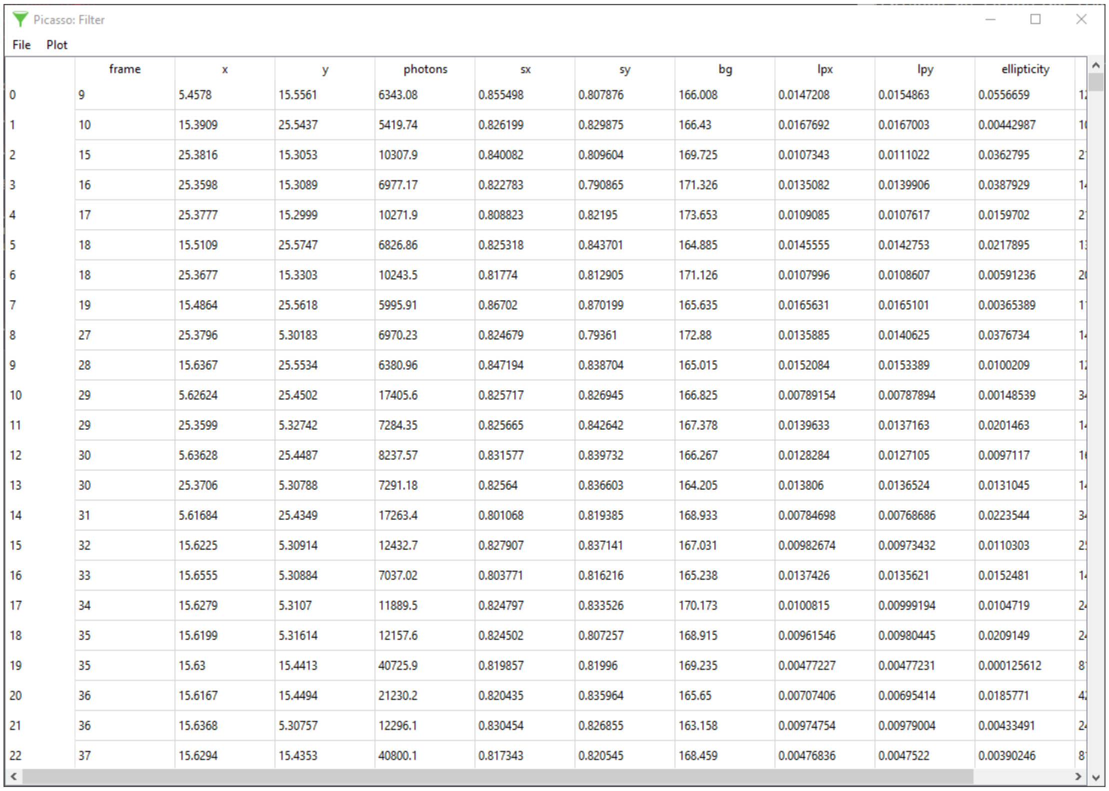

	frame	x	y	photons	sx	sy	bg	lpx	lpy	ellipticity
0	9	5.4578	15.5561	6343.08	0.855498	0.807876	166.008	0.0147208	0.0154863	0.0556659
1	10	15.3909	25.5437	5419.74	0.826199	0.829875	166.43	0.0167692	0.0167003	0.00442987
2	15	25.3816	15.3053	10307.9	0.840082	0.809604	169.725	0.0107343	0.0111022	0.0362795
3	16	25.3598	15.3089	6977.17	0.822783	0.790865	171.326	0.0135082	0.0139906	0.0387929
4	17	25.3777	15.2999	10271.9	0.808823	0.82195	173.653	0.0109085	0.0107617	0.0159702
5	18	15.5109	25.5747	6826.86	0.825318	0.843701	164.885	0.0145555	0.0142753	0.0217895
6	18	25.3677	15.3303	10243.5	0.81774	0.812905	171.126	0.0107996	0.0108607	0.00591236
7	19	15.4864	25.5618	5995.91	0.86702	0.870199	165.635	0.0165631	0.0165101	0.00365389
8	27	25.3796	5.30183	6970.23	0.824679	0.79361	172.88	0.0135885	0.0140625	0.0376734
9	28	15.6367	25.5534	6380.96	0.847194	0.838704	165.015	0.0152084	0.0153389	0.0100209
10	29	5.62624	25.4502	17405.6	0.825717	0.826945	166.825	0.00789154	0.00787894	0.00148539
11	29	25.3599	5.32742	7284.35	0.825665	0.842642	167.378	0.0139633	0.0137163	0.0201463
12	30	5.63628	25.4487	8237.57	0.831577	0.839732	166.267	0.0128284	0.0127105	0.0097117
13	30	25.3706	5.30788	7291.18	0.82564	0.836603	164.205	0.013806	0.0136524	0.0131045
14	31	5.61684	25.4349	17263.4	0.801068	0.819385	168.933	0.00784698	0.00768686	0.0223544
15	32	15.6225	5.30914	12432.7	0.827907	0.837141	167.031	0.00982674	0.00973432	0.0110303
16	33	15.6555	5.30884	7037.02	0.803771	0.816216	165.238	0.0137426	0.0135621	0.0152481
17	34	15.6279	5.3107	11889.5	0.824797	0.833526	170.173	0.0100815	0.00999194	0.0104719
18	35	15.6199	5.31614	12157.6	0.824502	0.807257	168.915	0.00961546	0.00980445	0.0209149
19	35	15.63	15.4413	40725.9	0.819857	0.81996	169.235	0.00477227	0.00477231	0.000125612
20	36	15.6167	15.4494	21230.2	0.820435	0.835964	165.65	0.00707406	0.00695414	0.0185771
21	36	15.6368	5.30757	12296.1	0.830454	0.826855	163.158	0.00974754	0.00979004	0.00433491
22	37	15.6294	15.4353	40800.1	0.817343	0.820545	168.459	0.00476836	0.0047522	0.00390246

5.1 Filtering of localizations

Open a localization HDF5 file in `Picasso: Filter` by dragging it into the main window or by selecting `File` > `Open`. The displayed table shows the properties of each localization in rows. Each column represents one property (e.g., coordinates, number of photons); see the filetypes section for details.

To display a histogram from values of one property, select the respective column in the header and select `Plot` > 'Histogram' (Ctrl + h). 2D histograms can be displayed by selecting two columns (press Ctrl to select multiple columns) and then selecting `Plot` > `2D Histogram` (Ctrl + d).

Left-click and hold the mouse button down to drag a selection area in a 1D or 2D histogram. The selected area will be shaded in green. Each localization event with histogram properties outside the selected area is immediately removed from the localization list.

Save the filtered localization table by selecting `File` > `Save`.

CHAPTER 6

render

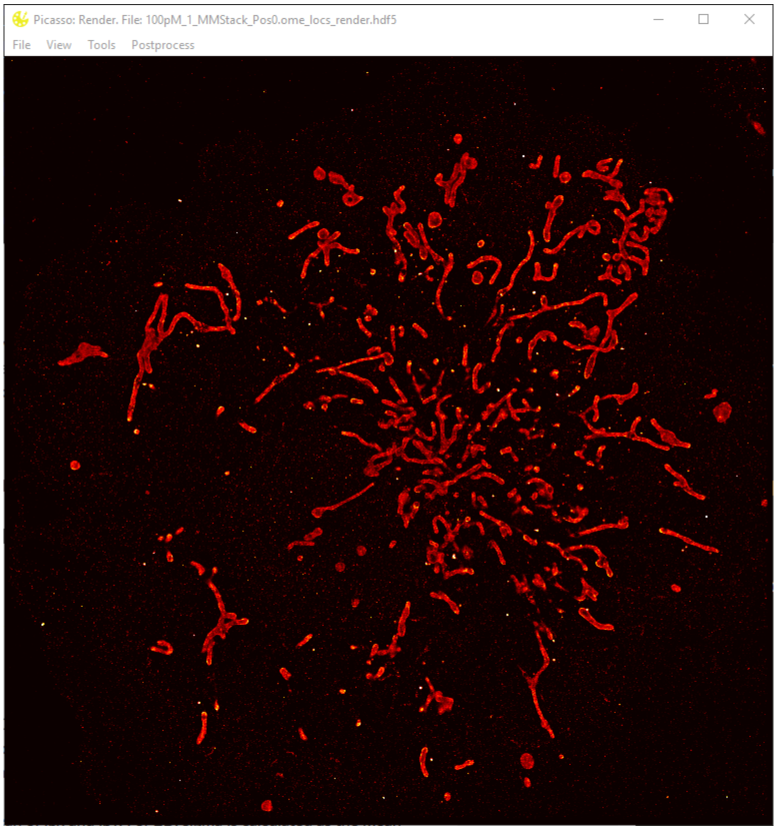

6.1 Opening Files

1. Rendering of the super-resolution image: In `Picasso: Render`, open a movie file by dragging a localization file (ending with '.hdf5') into the window or by selecting `File > Open`. The super-resolution image

will be rendered automatically. A region of choice can be zoomed into by a rectangular selection using the left mouse button. The 'View' menu contains more options for zooming and panning.

2. (Optional) Adjust rendering options by selecting `View > Display Settings`. The field 'Oversampling' defines the number of super-resolution pixels per camera pixel. The contrast settings `Min. Density` and `Max. Density` define at which number of localizations per super-resolution pixel the minimum and maximum color of the colormap should be applied.
3. (Optional) For multiplexed image acquisition, open HDF5 localization files from other channels subsequently. Alternatively, drag and drop all HDF5 files to be displayed simultaneously.

6.2 Drift Correction

Picasso offers two procedures to correct for drift: an RCC algorithm (option A), and use of specific structures in the image as drift markers (option B). Although option A does not require any additional sample preparation, option B depends on the presence of either fiducial markers or inherently clustered structures in the image. On the other hand, option B often supports more precise drift estimation and thus allows for higher image resolution. To achieve the highest possible resolution (ultra-resolution), we recommend consecutive applications of option A and multiple rounds of option B. The drift markers for option B can be features of the image itself (e.g., protein complexes or DNA origami) or intentionally included markers (e.g., DNA origami or gold nanoparticles). When using DNA origami as drift markers, the correction is typically applied in two rounds: first, with whole DNA origami structures as markers, and, second, using single DNA-PAINT binding sites as markers. In both cases, the precision of drift correction strongly depends on the number of selected drift markers.

6.2.1 Redundant cross-correlation drift correction

1. In `Picasso: Render`, select `Postprocess > Undrift by RCC`.
2. A dialog will appear asking for the segmentation parameter. Although the default value, 1,000 frames, is a sensible choice for most movies, it might be necessary to adjust the segmentation parameter of the algorithm, depending on the total number of frames in the movie and the number of localizations per frame. A smaller segment size results in better temporal drift resolution but requires a movie with more localizations per frame.
3. After the algorithm finishes, the estimated drift will be displayed in a pop-up window, and the display will show the drift-corrected image.

6.2.2 Marker-based drift correction

1. In `Picasso: Render`, pick drift markers as described in `Picking of regions of interest`. Use the `Pick similar` option to automatically detect a large number of drift markers similar to a few manually selected ones.
2. If the structures used as drift markers have an intrinsic size larger than the precision of individual localizations (e.g., DNA origami, large protein complexes), it is critical to select a large number of structures. Otherwise, the statistic for calculating the drift in each frame (the mean displacement of localization to the structure's center of mass) is not valid.
3. Select `Postprocess > Undrift from picked` to compute and apply the drift correction.
4. (Optional) Save the drift-corrected localizations by selecting `File > Save localizations`.

6.3 Picking of regions of interest

1. Manual selection. Open `Picasso: Render` and load the localization HDF5 file to be processed.
2. Switch the active tool by selecting `Tools > Pick`. The mouse cursor will now change to a circle. Alternatively, open `Tools > Tools Settings` to change the shape into a rectangle.
3. Set the size of the pick circle by adjusting the `Diameter` field in the tool settings dialog (`Tools > Tools Settings`). Alternatively, choose `Width` for a rectangular shape.
4. Pick regions of interest using the circular mouse cursor by clicking the left mouse button. All localizations within the circle will be selected for further processing.
5. (Optional) Automated region of interest selection. Select `Tools > Pick similar` to automatically detect and pick structures that have similar numbers of localizations and RMS deviation (RMSD) from their center of mass than already-picked structures. The upper and lower thresholds for these similarity measures are the respective standard deviations of already-picked regions, scaled by a tunable factor. This factor can be adjusted using the field `Tools > Tools Settings > Pick similar ± range`. To display the mean and standard deviation of localization number and RMSD for currently picked regions, select `View > Show info` and click `Calculate info below`.
6. (Optional) Exporting of pick information. All localizations in picked regions can be saved by selecting `File > Save picked localizations`. The resulting HDF5 file will contain a new integer column `group` indicating to which pick each localization is assigned.
7. (Optional) Statistics about each pick region can be saved by selecting `File > Save pick properties`. The resulting HDF5 file is not a localization file. Instead, it holds a data set called `groups` in which the rows show statistical values for each pick region.
8. (Optional) The picked positions and diameter itself can be saved by selecting `File > Save pick regions`. Such saved pick information can also be loaded into `Picasso: Render` by selecting `File > Load pick regions`.

6.4 Dialogs

6.4.1 Display Settings

Allows to change the display settings. Open via `View > Display Settings`.

General

Adjust the general display settings.

Zooom

Set the magnification factor.

Oversampling

Set the oversampling. Choose `dynamic` to automatically adjust to current window size when zooming.

Minimap

Click `show minimap` to display a minimap in the upper left corner to localize where the current field of view is within the image.

Contrast

Define the minimum and maximum density of the and select a colormap. Available colormaps are ['gray', hot', 'inferno', 'magma', 'plasma', 'viridis']. The selected colormap will be saved when closing render.

Blur

Select a blur method. Available options are: * None * One-Pixel-Blur * Individual Localization Precision * Individual Localization Precision, iso

Camera

Select the pixel size of the camera. This will be automatically set to a default value or the value specified in the *.yaml file.

Scale Bar

Activate scalebar. The length of the scale bar is calculated with the Pixel Size set in the Camera dialog. Activate `Print scale bar length` to additionally print the length.

Render properties

This allows rendering properties by color.

6.4.2 Show Info

Displays the info dialog.

Display

Shows the image width/height, the coordinates, and dimensions of the current FoV.

Movie

Displays the median fit precision of the dataset. Clicking on `Calculate` allows calculating the precision via the NeNA approach. See DOI: 10.1007/s00418-014-1192-3.

Field of view

Shows the number of localizations in the current FoV.

Picks

Allows calculating statistics about the picked localizations. Press `Calculate info below` to calculate. `Ignore dark times` allows treating consecutive localizations as on, even if there are localizations (specified by the parameter) missing between them. When defining the number of units per pick, you can calibrate the influx rate via `Calibrate influx`. A histogram of the dark and bright time can be plotted when clicking `Histograms`.

6.5 Menu items

6.5.1 File

Open [Ctrl+O]

Open an .hdf5 file to open in render.

Save localizations [Ctrl+S]

Save the localizations that are currently loaded in render to an hdf5 file.

Save picked localizations [Ctrl+Shift+S]

Save the localizations that are within a picked region (yellow circle or rectangle). Each pick will get a different group number. To display the group number in Render, select `Annotate picks` in Tools/Tools Settings. In case of rectangular picks, the saved localizations file will contain new columns *x_pick_rot* and *y_pick_rot*, which are localization coordinates into the coordinate system of the pick rectangle (coordinate (0,0) is where the rectangle was started to be drawn, and *y_pick_rot* is in the direction of the drawn line.) These columns can be used to plot density profiles of localizations along the rectangle dimensions easily (e.g., with “Filter”).

Save pick properties

Calculates the properties of each pick (i.e., mean frame, mean x mean y as well as kinetic information and saves it as an hdf5 file.

Save pick regions

Saves the positions of the picked regions (yellow circles) in a .yaml file. The file will contain the following: A list of center positions and the value of the diameter. It is possible to manually add center positions or copy from another pick regions file with a text editor.

Load pick regions

Resets the current picked regions and loads regions from a .yaml file that contains pick regions.

Export ROI for Imaris

This function allows to export the current ROI for Imaris. Note that this is currently only implemented for Windows. Click on File / Export ROI for imaris and enter a filename for export. Picasso will export the current region of interest with the current oversampling settings. If multiple channels are loaded it will export the channels with the same colors as set in Picasso (Shortcut CTRL+F or View / Files to change.) Depending on the size of the ROI, the export will take a couple of seconds. Once exporting is finished, the file will be saved at the set location. The resulting file can be opened e.g. with ImarisViewer or Imaris. Note that the orientation is the same as in Picasso.

Export localizations

Select export for various other programs. Note that some exporters only work for 3D files (with z coordinates). For additional file converters check out the convert folder at Picasso's GitHub page.

Export as .csv for ThunderSTORM

This will export the dataset in a .csv file to use with ThunderSTORM.

Note that for large datasets the writing of the file may take some time.

Note that the pixel size value that is set in Display Settings will be used for exporting.

Thefollowing columns will be exported: 3D: id, frame, x [nm], y [nm], z [nm], sigma1 [nm], sigma2 [nm], intensity[photon], offset[photon], uncertainty_xy [nm] 2D: id, frame, x [nm], y [nm], sigma [nm], intensity [photon], offset [photon], uncertainty_xy [nm]

The uncertainty_xy is calculated as the mean of lpx and lpy. For 2D, sigma is calculated as the mean of sx and sy.

For the case of linked localizations, a column named `detections` will be added, which contains the len parameter - that's the duration of a blinking event and not the number n of linked localizations. This is meant to be better for downstream kinetic analysis. For a gradient that is well-chosen n ~ len and for a gap size of 0 len = n.

Export as .txt for FRC

Export as .txt file to be used for the fourier ring correlation plugin in ImageJ.

Export as .xyz for Chimera

Export as .txt file to be used for Chimera import.

Export as .3d for ViSP

Export as .3d file to be used ViSP.

6.5.2 View

Display settings (CTRL + D)

Opens the Display Settings Dialog.

Files (CTRL + F)

Open a dialog to select the color and toggle visibility for each loaded dataset.

Left / Right / Up / Down

Moves the current field of view in a particular direction. Also possible by using the arrow keys.

Zoom in (CTRL +)

Zoom into the image.

Zoom out (CTRL -)

Zoom out of the image.

Fit image to window

Fits the reconstructed image to be fully displayed in the window.

Slice (3D)

Opens the slicer dialog which allows for slicing through 3D datasets.

Show info

Shows info for the current dataset. See Info Dialog.

6.5.3 Tools

Zoom (CTRL + Z)

Selects the zoom tool. The mouse can now be used for zoom and pan.

Pick (CTRL + P)

Selects the pick tool. The mouse can now be used for picking localizations. The user can set the pick shape in the *Tools settings* (CTRL + T) dialog. The default shape is Circle with the diameter to be set. For rectangles, the user draws the length, while the width is controlled via a parameter for all drawn rectangles, similar to the diameter for circular picks.

Measure (CTRL + M)

Selects the measure tool. The mouse can now be used for measuring distances. Left click adds a crosshair for measuring; right-click deletes the last crosshair.

Tools settings (CTRL + T)

Define the settings of the tools, i.e., the radius of the pick and an option to annotate each pick. For the circular picks the range of pick similar can be set.

Pick similar (CTRL + Shift + P)

Automatically identifies picks that are similar to the current picks.

Show trace (CTRL + R)

Shows the time trace of the currently selected pick(s).

Select picks (trace)

Opens a dialog to that goes through all picks, displays its trace and asks to keep or discard it.

Select picks (XY scatter)

Opens a dialog to that goes through all picks, displays a xy-scatterplot and asks to keep or discard it.

Plot pick (XYZ scatter) (CTRL + 3)

Displays a 3D scatterplot of the localizations of the currently selected pick(s).

Select picks (XYZ scatter)

Opens a dialog to that goes through all picks, displays an xyz-scatterplot and asks to keep or discard it.

Select picks (XYZ scatter, 4 panels)

Opens a dialog to that goes through all picks, displays four panels with an xyz-scatterplot and a top, bottom and side projection and asks to keep or discard it.

Filter picks by locs

Allows filtering picks by the number of localizations in each pick. When clicking, a histogram of the number of localizations of all selected picks will be calculated. A lower and upper boundary can be selected to filter the picks.

Clear picks (Ctrl + C)

Clears all currently selected picks.

Subtract pick regions

Allows loading another pick regions file to subtract from the currently selected picks. Can be slow for a large number of picks.

Show FRET traces

Allows showing FRET traces for picks. This requires to have an acceptor and donor dataset loaded. Both channels should be aligned (i.e., via the `Align channels (RCC or from picked)` function). `Show FRET traces` will calculate a FRET intensity when two single-molecule events in one pick occur in the same frame and display a trace for these events. The intensity is calculated as I = I_A/(I_A+I_D). Here, I_A and I_D are the photon values of the localization minus the calculated background. Only FRET events > 0 and < 1 will be displayed.

Calculate FRET in picks

Allows calculating FRET for several picks. This requires to have an acceptor and donor dataset loaded. Both channels should be aligned (i.e., via the Align channels function). The FRET intensity is calculated when two single-molecule events in one pick occur in the same frame. The intensity is calculated as I = I_A/(I_A+I_D). Here, I_A and I_D are the photon values of the localization minus the calculated background. Only FRET events in a range of > 0 and < 1 are kept.

After calculation, a histogram of the FRET intensities is displayed. Additionally, all localizations with a valid FRET intensity are saved in an hdf5 file. The localizations have an additional column with the FRET intensities. This allows reloading the FRET-localizations in render. To color-code for FRET-intensity, use the render properties function and select FRET. Additionally, a txt document is saved containing a list of the FRET values as it was used to display the histogram.

Note: In order to calculate meaningful FRET data, the selected picks should contain data in the donor and acceptor channel. To ensure this, a sample workflow could be as follows: - Align the channels via `Align channels (RCC or from picked)` - Pick some regions in one channel (i.e., the donor channel) - Calculate the pick properties - Adjust the `Pick similar` parameter accordingly and pick similar - Filter in the other channel (i.e., the acceptor channel) via `Filter picks by locs` to have at least a minimum number of localizations - Use the calculate FRET in picks function

Cluster in pick (k-means)

Allows performing k-means clustering in picks. Users can specify the number of clusters and deselect individual clusters. Picks can be kept or removed. After looping through all picks an hdf5 file with the cluster information can be saved.

Mask image

6.5.4 Postprocess

Undrift by RCC

Performs drift correction by redundant cross-correlation.

Undrift from picked (3D)

Performs drift correction using the picked localizations as fiducials. Also performs drift correction in z if the dataset has 3D information.

Undrift from picked (2D)

Performs drift correction using the picked localizations as fiducials. Does not perform drift correction in z even if dataset has 3D information.

Undo drift (2D)

Undo previous drift correction (only 2D part). Can be pressed again to redo.

Show drift

After drift correction, a drift file is created. If the drift file is present, the drift can be displayed with this option.

Remove group info

Removes the group information when loading a dataset that contains group information. This will, i.e., turn the multicolor representation into a single color representation.

Unfold / Refold groups

Allows to "unfold" an average to display each structure individually in a line. Also works with picks.

Unfold groups (square)

Arranges an average in a square so that each structure is displayed individually

Link localizations

Links consecutive localizations

Align channels (RCC or from picked)

Aligns channels to each other when several datasets are loaded. If picks are selected, the alignment will be via the center of mass of the picks; otherwise, an RCC will be used.

Combine locs in picks

Combines all localizations in each pick to one.

Apply expressions to localizations

This tool allows you to apply expressions to localizations.

dbscan

Cluster localizations with the dbscan clustering algorithm.

hdbscan

Cluster localizations with the hdbscan clustering algorithm.

Examples

- `x +=1` will shift all localization by one to the right
- `x +=1;y+=1` will shift all localization by one to the right and one up.

Notes

Using two variables in one statement is not supported (e.g. `x = y`) To filter localizations use picasso filter.

Additional commands

`flip x z` will exchange the x-axis with y-axis if z localizations are present (side projection), similar for `flip y z`. `spiral r n` will plot each localization over the time of the movie in a spiral with radius r and n number of turns (e.g., to detect repetitive binding), `uspiral` to reverse.

CHAPTER 7

average

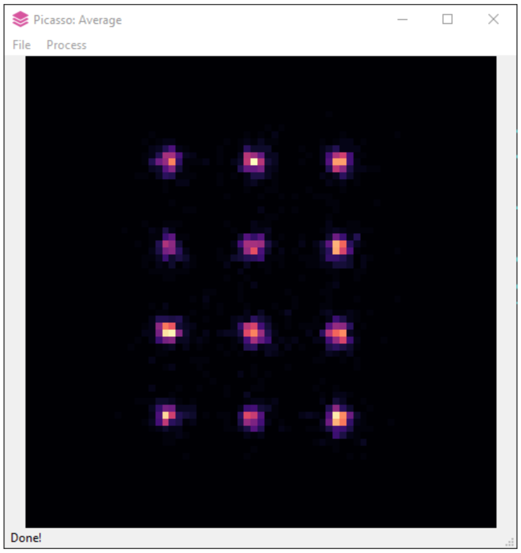

7.1 Particle Averaging

The averaging module uses 2D cross-correlation to determine the rotational and translational offset.

1. In `Picasso: Render`, pick structures to be averaged.
2. Save the picked localizations by selecting `File` > `Save picked localizations`.
3. Load the resulting file with picked localizations into `Picasso: Average` by selecting `File` > `Open` or dragging and dropping it into the window. It will start a parallel pool to increase computational speed.
4. `Picasso: Average` will immediately perform a center-of-mass alignment of the picked structures and display an average image. Rotational and translational alignment will follow in the next steps.

5. Select `Process` > `Parameters` and adjust the `Oversampling` parameter. We recommend choosing the highest number at which the average image still appears smooth. High oversampling values result in substantial computational time and can cause artifacts. Hence, it might be useful to first use low oversampling to generate a less-refined average image and then perform subsequent averaging steps with higher oversampling for optimized resolution.
6. Adjust the number of average iterations in the `Iterations` field. In most cases, a value of 10 is more than sufficient. If you are unsure about the computational time of the process, choose one iteration as a starting point. More iterations can be added later by repeating the processing steps. After a certain number of iterations, the average image will converge, meaning that it will not change with more iterations. If you experience that one localization spot is overemphasized, try again with less oversampling and few iterations. The 2D cross-correlation is prone to lock in bright spots.
7. Select `Process` > `Average` to perform particle averaging with the current oversampling for the set number of iterations. This step can be repeated with different settings. The program will use the current average image as a starting point.
8. Once the average image has converged, save the transformed localizations by selecting `File` > `Save`. The resulting HDF5 localization file contains the aligned localizations in the center of the movie dimensions. It can be loaded like any other HDF5 localization file into `Picasso: Render`.

CHAPTER 8

filetypes

This section describes the different file format and name conventions used in Picasso.

8.1 Movie Files

Picasso accepts two types of raw movie files: TIFF (preferably from μManager1) and raw binary data (file extension ".raw").

When loading raw binary files, the user will be prompted for movie metadata such as the number of frames, number of pixels, etc. Alternatively, this metadata can be supplied by an accompanying metadata file with the same filename as the raw binary file, but with the extension .yaml. See `YAML Metadata Files` for more details.

8.2 HDF5 Files

HDF5 is a generic and efficient binary file format for storing data2. In Picasso, HDF5 files are used for storing tabular data of localization properties with the file extension .hdf5. Furthermore, Picasso saves the statistical properties of groups of localizations in an HDF5 file.

Generally, several datasets can be stored within an HDF5 file. These datasets are accessible by specifying a path within the HDF5 file, similar to a path of an operating system. When saving localizations, Picasso stores tabular data under the path `/locs`. When saving statistical properties of groups of localizations, Picasso saves the table under the path `/groups`.

8.3 Importing HDF5 files in Pandas, MATLAB and Origin

In Pandas, use pandas.read_hdf().

In MATLAB, execute the command `locs = h5read(filename, dataset)`. Replace dataset with `/locs` for localization files and with `/groups` for pick property files.

In Origin, select `File > Import > HDF5` or drag and drop the file into the main window.

8.4 Localization HDF5 Files

Localization HDF5 files must always be accompanied by a YAML metadata file with the same filename, but with the extension .yaml. See `YAML Metadata File` for more details. The localization table is stored as a dataset of the HDF5 file in the path `/locs`. This table can be visualized by opening the HDF5 file with `Picasso: Filter`. The localization table can have an unlimited number of columns. Table 1 describes the meaning of Picasso's main column names.

Table 1: Table 1: Name, description and data type for the main columns used in Picasso.

Column Name	Description	C Data Type
frame	The frame in which the localization occurred, starting with zero for the first frame.	unsigned long
x	The subpixel x coordinate in camera pixels	float
y	The subpixel y coordinate in camera pixels	float
photons	The total number of detected photons from this event, not including background or camera offset	float
sx	The Point Spread Function width in camera pixels	float
sy	The Point Spread Function height in camera pixels	float
bg	The number of background photons per pixel, not including the camera offset	float
lpx	The localization precision in x direction, in camera pixels, as estimated by the Cramer-Rao Lower Bound of the Maximum Likelihood fit.	float
lpy	The localization precision in y direction, in camera pixels, as estimated by the Cramer-Rao Lower Bound of the Maximum Likelihood fit.	float
net_gradient	The net gradient of this spot which is defined by the sum of gradient vector magnitudes within the fix box, projected to the spot center.	float
likelihood	The log-likelihood of the fit	float
iterations	The number of iterations of the fit procedure	long
group	(Optional) An identifier to assign multiple localizations to groups, for example by picking regions of interest	long
len	(Optional) The length of the event, if localizations from consecutive frames have been linked	long
n	(Optional) The number of localizations in this event, if localizations from consecutive frames have been linked, potentially diverging from the "len" column due to a transient dark time tolerance	long
photon_rate	(Optional) The mean number of photons per frame, if localizations from consecutive frames have been linked. The total number of photons is set in the "photons" column.	float

8.5 HDF5 Pick Property Files

When selecting `File > Save pick properties` in `Picasso: Render`, the properties of picked regions are stored in an HDF5 file. Within the HDF5 file, the data table is stored in the path `/groups`. Each row in the "groups" table corresponds to one picked region. For each localization property (see Table 1), two columns are generated in the `groups` table: the mean and standard deviation of the respective column over the localizations in a pick region. For example, if the localization table contains a column `len`, the "groups" table will contain a column `len_mean` and `len_std`. Furthermore, the following columns are included: `group` (the group identifier), `n_events` (the number of localizations in the region) and `n_units` (the number of units from a qPAINT measurement).

8.6 YAML Metadata Files

YAML files are document-oriented text files that can be opened and changed with any text editor3. In Picasso, YAML files are used to store metadata of movie or localization files. Each localization HDF5 file must always be accompanied with a YAML file of the same filename, except for the extension, which is `.yaml`. Deleting this YAML metadata file will result in failure of the Picasso software! Raw binary files may be accompanied by a YAML metadata file to store data about the movie dimensions, etc. While the metadata file, in this case, is not required, it reduces the effort of typing in this metadata each time the movie is loaded with `Picasso: Localize`. To generate such a YAML metadata file, load the raw movie into `Picasso: Localize`, then enter all required information in the appearing dialog. Check the checkbox `Save info to yaml file` and click ok. The movie will be loaded and the metadata saved in a YAML file. This file will be detected the next time this raw movie is loaded, and the metadata does not need to be entered again.a

CHAPTER 9

CMD

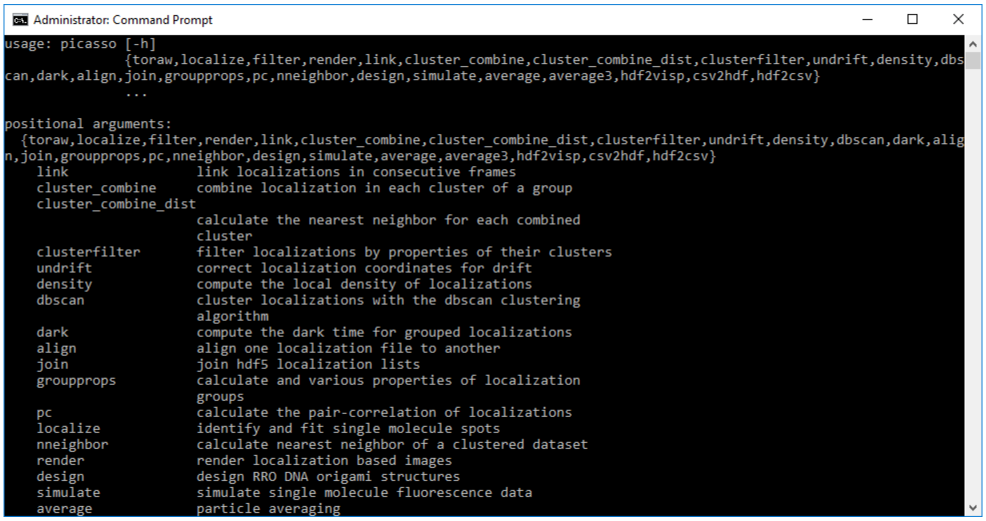

Here is a list of command-line commands that can be used with picasso.

9.1 localize

Reconstructing images via command line is possible. Type: `python -m picasso localize args` within an environment or `picasso localize args` if Picasso is installed.

9.1.1 Batch process a folder

To batch process a folder simply type the folder name (or drag in drop into the console), e.g. `python -m picasso localize foldername`. Picasso will analyze the folder and process all *.ome.tif in files in the folder. If the files have consecutive names (e.g., File.ome.tif, File_1.ome.tif, File_2.ome.tif), they will be treated as one.

If you want to analyze *.raw files, Picasso will check whether a *.raw file has a corresponding *.yaml file. If none is found, you can enter the specifications for each raw file. It is possible to use the same specifications for all *.raw files in that run.

Adding additional arguments

The reconstruction parameters can be specified by adding respective arguments. If they are not specified the default values are chosen.

```
'-b', '-box-side-length', type=int, default=7, help='box side length'
'-a', '-fit-method', choices=["mle", "lq", "lq-gpu", "lq-3d", "lq-gpu-3d", "avg"],␣
↪default='mle'
'-g', '-gradient', type=int, default=5000, help='minimum net gradient'
'-d', '-drift', type=int, default=1000, help='segmentation size for subsequent RCC, 0␣
↪to deactivate'
'-bl', '-baseline', type=int, default=0, help='camera baseline'
'-s', '-sensitivity', type=int, default=1, help='camera sensitivity'
'-ga', '-gain', type=int, default=1, help='camera gain'
'-qe', '-qe', type=int, default=1, help='camera quantum efficiency'
```

Note 1: Localize will automatically try to perform an RCC drift correction on the dataset. As this will not always work with the default settings after an unsuccessful attempt, the program will continue with the next file. If the drift correction succeeds, another hdf5 file with the drift corrected locs will be created.

Note 2: Make sure to set the camera settings correctly; otherwise Photon counts are wrong plus the MLE might have problems.

Note 3: If you select one of the 3D algorithms (lq-3d or lq-gpu-3d) the program will ask you to enter the magnification factor and the path to the 3D calibration file.

Example

This example shows the batch process of a folder, with movie ome.tifs that are supposed to be reconstructed and drift corrected with the `lq`-Algorithm and a gradient of 4000.

`python -m picasso localize foldername -a lq -g 4000` or `picasso localize foldername -a lq -g 4000`

9.2 csv2hdf

Convert csv files (thunderSTORM) to hdf. Type `python -m picasso csv2hdf filepath pixelsize`. Note that the following columns need to be present: `frame, x_nm, y_nm, sigma_nm, intensity_photon, offset_photon, uncertainty_xy_nm` for 2D files `frame, x_nm, y_nm, z_nm, sigma1_nm, sigma2_nm, intensity_photon, offset_photon, uncertainty_xy_nm` for 3D files

9.3 join

Combine two hdf5 localization files. Type `python -m picasso join file1 file2`. A new joined file will be created. Note that the frame information is preserved, i.e., frame 1 now can contain localizations from file 1 and file 2. Therefore, do not perform kinetic analysis and drift correction on joined files.

9.4 link

Link localizations in consecutive frames.

9.5 cluster_combine

Combines the localizations in each cluster of a group.

9.6 cluster_combine_dist

Calculate the nearest neighbor for each combined cluster

9.7 clusterfilter

Filter localizations by properties of their clusters.

9.8 undrift

Correct localization coordinates for drift with RCC.

9.9 density

Compute the local density of localizations

9.10 dbscan

Cluster localizations with the dbscan clustering algorithm.

9.11 hdbscan

Cluster localizations with the hdbscan clustering algorithm.

9.12 dark

Compute the dark time for grouped localizations.

9.13 align

Align one localization file to antoher via RCC. Type `python -m picasso align file1 file2`

9.14 groupprops

Calculate the properties of localization groups

9.15 pc

Calculate the pair-correlation of localizations

9.16 nneighbor

Calculate the nearest neighbor within a clustered dataset

9.17 render

Start the render module

9.18 design

Start the design module.

9.19 simulate

Start the simulation module.

9.20 average

Start the 2D averaging module

9.21 average3

Start the 3D averaging module

CHAPTER 10

Postprocessing

10.1 Jupyter Notebooks

For additional postprocessing steps, one might want to use different libraries without having to install the picasso package. For this check out the samples folder. Here you can find sample jupyter notebooks that show how the Picasso package can be used.

10.1.1 SampleNotebook

This notebook shows some basic interaction with the .hdf5 files using the pandas library.

10.1.2 SampleNotebook1

This notebook shows some basic interaction with the picasso library, e.g. how to directly call functions from a Jupyter Notebook.

10.1.3 SampleNotebook2

This notebook shows how to perform HDBSCAN clustering with picasso.

CHAPTER 11

nanoTRON

The training module uses scikit-learn 's multi-layer perceptron (MLP) to train an artificial neural network (ANN). If you use nanoTRON, please cite the publication:

- A Auer, M T Strauss, S Strauss, and R Jungmann. "nanoTRON: a Picasso module for MLP-based classification of super-resolution data". Bioinformatics, 2020. doi: 10.1093/bioinformatics/btaa154

Detailed recommendation for the use of nanoTRON can be found in the supplementary information, here.

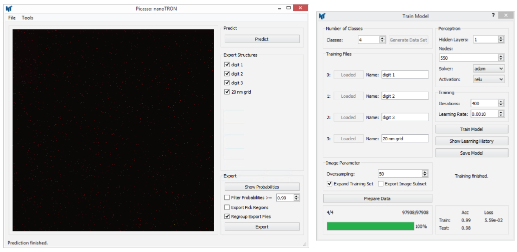

11.1 nanoTRON Train

Train a MLP model for nanopattern prediction using nanoTRON.

1. In `Picasso: nanoTRON`, select `Tools` > `Train Model` to open the training user interface.
2. Set the number of different classes of the model via `Number of Classes` and press `Generate Data Set`.
3. Load all the training files via the various buttons `Load File`. Greyed out buttons indicate that the data set slot is loaded.
4. Name every data set (class) with a unique name.
5. Set up the image parameters in the box `Image Parameter`. Set the oversampling factor via `Oversampling`. This factor determines the sub-pixel resolution of the training images. We recommend lower resolution as the data would provide for better generalization. Augment the training data sets via the

check button `Expand Training Set`. Here every data set point is rotated eleven times. Exemplary images training data images can be exported via `Export Image Subset`.

6. If the data is loaded and the image parameters are set, press `Prepare Data` to convert the localization (molecule) tables into images.
7. Set up the MLP in the box `Perceptron` Set the number of hidden layers via `Hidden Layers`. The number of nodes in every layer can be set via `Nodes`. Choose the type of training algorithm via the dropdown box `Solver`. We recommend using `adam`. Choose the activation function of the layer via the dropdown box `Activation`. Note, the activation function can only be set for the whole network.
8. Set up the training in the box `Training`. Set the maximum number of iterations (epochs) via the box `Iterations`. Set the learning rate via `Learning Rate` (only necessary if SGD solver is chosen).
9. To start the training, press `Train Model`. Depending on the CPU and the size of the training data, this can take up to a few hours.
10. When the training is finished, the learning curve and the confusion matrix can be plotted via `Show Learning History`.
11. A summary of the achieved train and test accuracies is given at the bottom of the `Train Model` window.
12. Save the model for later use via `Save Model`.

11.2 nanoTRON Predict

Use a trained nanoTRON model to classify nanopatterns on new data.

1. Load the model via `Tools` > `Train Model`. The different classes should now be listed on the right side.
2. Drag and drop the Picasso localization table file (HDF5) that should be used for prediction into the large grey box. If the file was loaded correctly, the box displays the image.
3. Start the classification via the button `Predict`.
4. Choose the classes that should be exported in the box `Export Structures`. By default, each class is exported.
5. Set the export parameters in the box `Export`. The distribution of the prediction probability can be plotted via the button `Show Probabilities`. Optional, set a probability filter via the check button `Filter Probabilities`. Optional, enable the additional export of the pick regions via the check button `Export Pick Regions`. Choose if the new localization files, segmented by the classified nanopatterns, should be regrouped via the check button `Regroup Export Files`. Note, the group id in every exported file start with 0 then. Identification of picks in the original file will be lost.

CHAPTER 12

FAQ

12.1 Localize

12.1.1 What doest MLE / LQ stand for?

- MLE, integrated Gaussian (based on Smith et al., 2014.)
- LQ, Gaussian (least squares)

12.1.2 Picasso freezes during the localization step

This was a bug that could occur in the old picasso versions and should be fixed with version 0.2.4.

If it is always at the same localization:

- Try fitting with the LQ method; it tends to be more robust
- Try increasing the gradient
- Make sure to set the correct camera parameters (Baseline etc.)

CHAPTER 13

Indices and tables

- genindex
- modindex
- search
